# Tuberculosis in a Liver Cirrhosis Patient: A Management Conundrum

**DOI:** 10.7759/cureus.53533

**Published:** 2024-02-04

**Authors:** Pradnya M Diggikar, Hansini R Reddy, Mayank Mundada, Tushar Pancholi, Ahsan A Faruqi

**Affiliations:** 1 General Medicine, Dr. D. Y. Patil Medical College, Hospital & Research Centre, Pune, IND

**Keywords:** tuberculosis (tb), serum transaminases, inhomogeneous opacity, drug-induced liver injury (dili), liver function test (lft), att-related hepatoxicity, immunosuppression, child turcotte pugh, cirrhosis

## Abstract

This case report delves into the intricate challenges of managing tuberculosis (TB) in a 70-year-old male with decompensated chronic liver disease (DCLD) and a history of endoscopic variceal ligation. The patient, initially presenting with symptoms such as black-colored stools, breathlessness, and weight loss, was diagnosed with right-sided pneumonia alongside DCLD. Despite the administration of standard beta-lactam plus macrolide antibiotics, the patient exhibited no improvement. Subsequent bronchoscopy revealed *Mycobacterium tuberculosis* (MTB), prompting the initiation of first-line anti-tubercular therapy. However, the hepatotoxic response necessitated a switch to a modified regimen with non-hepatotoxic drugs, emphasizing the challenge of managing TB in cirrhotic patients. Effective management of MTB infection involves personalized administration of anti-TB drugs, taking into account the individual's chronic liver disease status. This case underscores the importance of treating tuberculosis in liver cirrhosis patients based on the Child-Turcotte-Pugh score. A tailored and vigilant approach is indispensable for the successful management of MTB infection.

## Introduction

Tuberculosis (TB) continues to pose a significant public health challenge in India due to its persistent morbidity and mortality burden, making it a chronic infectious disease of concern [1]. TB primarily impacts the lungs, but extrapulmonary cases can occur in 15-20% of patients [2]. The 2019 WHO Global TB report revealed that approximately 10 million people worldwide contracted TB before the COVID-19 pandemic [3]. The global burden of disease study in 2017 estimated 112 million compensated cirrhosis cases globally, resulting in an age-standardized global prevalence of 1,395 cases per 100,000 individuals. Cirrhosis was linked to 2.4% of fatalities worldwide in 2019 due to immunosuppression and an increased susceptibility to infections like TB [4]. Studies show that cirrhotic individuals have a 15-fold higher prevalence of TB than the general population, especially in West India. Another study conducted in India found that the prevalence of TB was almost five times higher in cirrhotic patients (8.1%) than in the general population (1.6%) [5]. Autopsy studies indicate a common occurrence of liver cirrhosis (LC), with a global prevalence of 5-10% in the general population [6]. The elevated risk of TB is attributed to immune dysfunction and the heightened virulence associated with cirrhosis. Over 90 percent of TB cases result from the reactivation of latent forms typically found in individuals with some degree of immunodeficiency or immunosuppression [7]. Given that cirrhosis is inherently an immunodeficient state, it is reasonable to assert that the prevalence of TB is significantly higher in cirrhotic individuals compared to the general population. The incidence of TB in cirrhotic patients was found to be 168.6 per 100,000 in a Danish cohort study conducted from 1977 to 1993. In men over the age of 65, the incidence peaked at 246 per 100,000. Additionally, cirrhotic patients who acquire TB face a poor prognosis [7]. Treating TB in patients with liver disease presents numerous clinical challenges. Managing individuals with underlying disorders is complex, involving issues such as poor tolerance, an increased incidence of hepatotoxicity, and significant alterations in the liver function. The variables impact drug pharmacokinetics and increase the risk of tuberculosis developing resistance to multiple drug treatments. Moreover, there is a higher likelihood of drug-induced liver damage, particularly in individuals with more advanced cirrhosis [8]. Here, we present a case emphasizing the challenges of treating pulmonary TB in a cirrhosis patient, underscoring the necessity for a standard protocol to streamline the management of TB in chronic liver disease patients and for monitoring anti-tubercular therapy (ATT)-related hepatoxicity.

## Case presentation

A 70-year-old male presented with complaints of black-colored stools persisting for three months, grade three breathlessness as per the modified Medical Research Council Dyspnea scale, and a non-productive cough lasting for 20 days. The patient reported a weight loss of 10kg in two months, associated with generalized weakness and loss of appetite. There was no history of fever. The patient has been diagnosed with decompensated chronic liver disease (DCLD) for the past 10 years, with a history of undergoing endoscopic variceal ligation (EVL) banding five times since 2012. The last EVL banding was done five months ago. He had a history of alcohol consumption for 30 years about 180ml per day. His last intake was in 2012.

**Figure 1:**
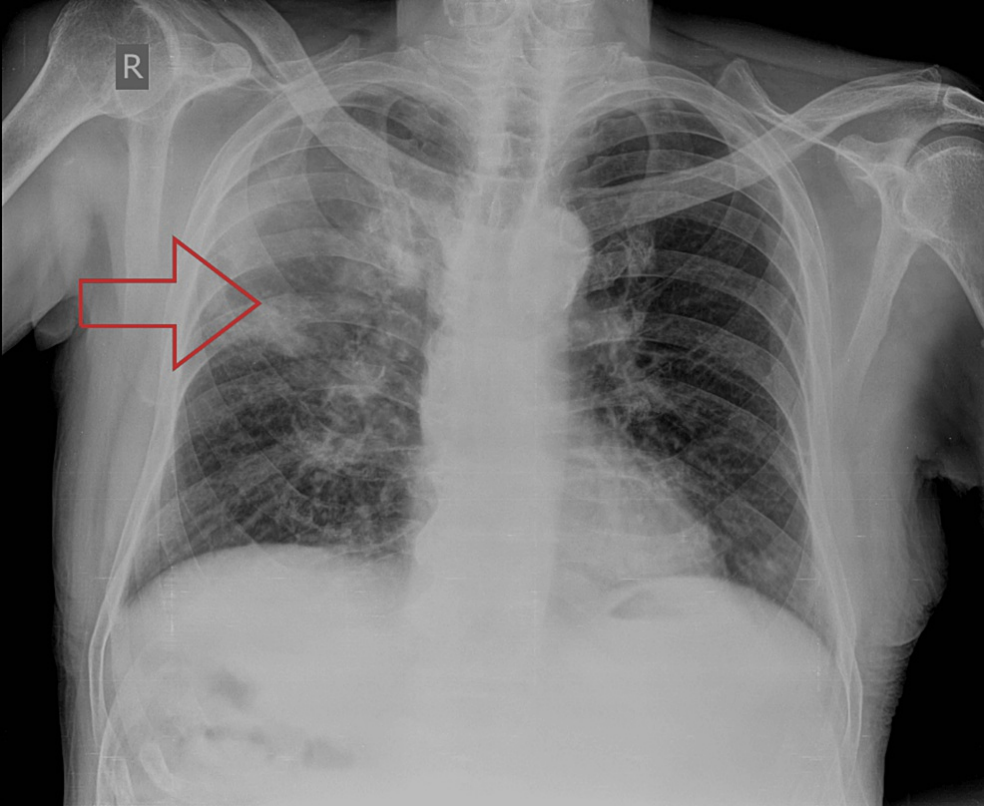
Chest X-ray PA view showing inhomogeneous opacities in the right upper lobe (red arrow) PA: posteroanterior

Clinical examination revealed pallor and pitting edema in both lower limbs. Vital signs were stable, and the cardiovascular examination was unremarkable. On per-abdomen palpation, a soft abdomen without tenderness or organomegaly was noted. Respiratory examination revealed normal vesicular breath sounds with decreased air entry in the right infraclavicular region and supraclavicular region and an impaired note on percussion of these regions suggestive of right-sided pneumonia. A chest X-ray was done which revealed inhomogeneous opacities in the right upper lobe (Figure 1). 

Abdominal ultrasound indicated chronic liver parenchymal disease, mild periportal cuffing, and mild ascites. Upper gastrointestinal endoscopy revealed three columns of large esophageal varices with a red color sign, post-EVL scarring, severe portal hypertensive gastropathy, and gastric antral vascular ectasia (GAVE). EVL banding was done on three esophageal varices.

**Figure 2:**
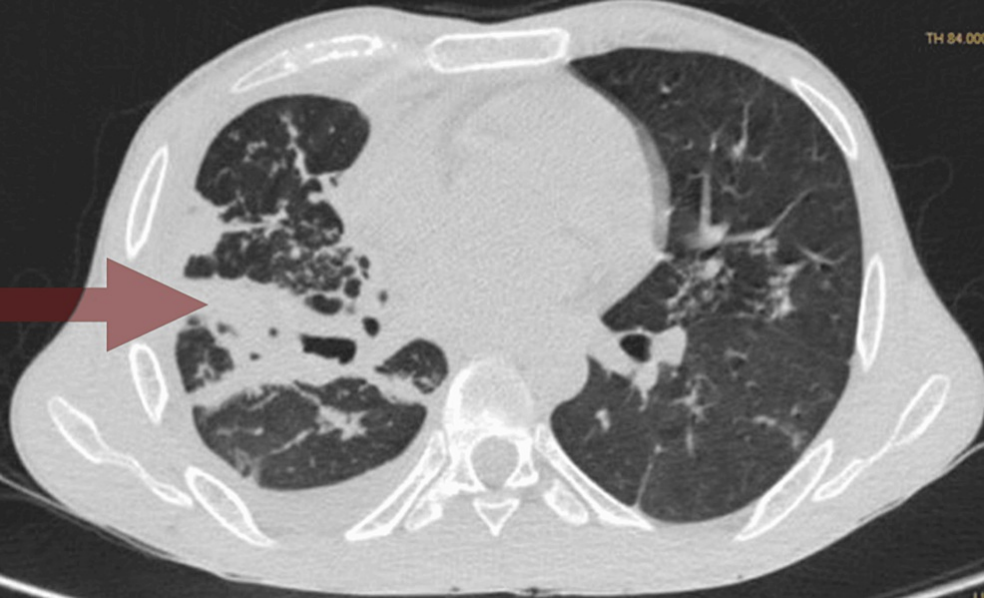
HRCT thorax in axial view showing ground glass opacities with consolidation (red arrow) in the right upper lobe HRCT: High-resolution computed tomography

Initial investigations (Table 1) indicated anemia of chronic disease, hyponatremia, and hypoalbuminemia, resulting in a class B Child-Turcotte-Pugh (CTP) score. High-resolution computed tomography (HRCT) (Figure 2) revealed multiple patchy ground-glass opacities with consolidation in the right upper and middle lobes, along with multiple sub-centimetric lymph nodes with few of them calcified.

**Table 1:** Laboratory Investigations on the day of admission MCV: Mean corpuscular volume; PCV: packed cell volume; CRP: c reactive protein; ESR: erythrocyte sedimentation rate; INR: international normalized ratio; MELD-Na: model for end-stage liver disease-sodium; HIV: human immunodeficiency virus; HbSAg: hepatitis B surface antigen; Anti HCV Ab: anti-hepatitis C antibody.

Parameter	Report	Normal limit
Hemoglobin	8	13.2-16.6gm/dl
Total leukocyte count	10,400	4000-10,000/micro L
Platelet count	88,000	1,50,000-4,10,000/micro L
PCV	33.9	38.30 - 48.60 %
MCV	100	78.2 - 97.9 fL
Total bilirubin	1.84	0.22-1.2 mg/dL
Direct bilirubin	1.11	up to 0.5
Alanine transaminases	13	7-55IU/L
Aspartate transaminases	20	8-48 IU/L
Alkaline phosphatase	64	40-129 U/L
Serum Urea	53	17-49mg/dL
Serum Creatinine	1.19	0.6-1.35mg/dL
Serum Sodium	128	136-145 mmol/L
Serum Potassium	4	3.5-5.1 mmol/L
Serum Chloride	99	98-107 mmol/L
Total protein	6.5	6.4-8.3 g/dL
Serum Albumin	2.5	3.5-5.2 g/dL
Serum ESR	25	up to 20mm/hr
Serum CRP	45.7	up to 5 mg/L
HIV/ HbSAg/ HCV	Non-reactive	non-reactive
Prothrombin Time	19.2	10.09-13.79 seconds
INR	1.8	0.85-1.15
Stool occult blood	occult blood positive	occult blood negative
Blood ammonia	35	20-120 microgram/dL
Urine routine microscopy	Within normal limits	within normal limits
Child-Turcotte-Pugh score	Class B (9 points)	5-15 points
Meld-Na	24 points	6-40 points

The patient was diagnosed with right-sided pneumonia alongside DCLD with portal hypertension and post-EVL banding status. Initially, beta-lactam plus macrolide antibiotics were administered, along with symptomatic treatment for DCLD. However, the patient showed no symptomatic improvement. Subsequently, bronchoscopy with bronchoalveolar lavage (BAL) was performed, and the sample was sent for cartridge-based nucleic acid amplification testing, yielding a positive result for *Mycobacterium tuberculosis *(MTB) with rifampicin (RIF) sensitivity. The patient was then initiated on first-line antitubercular therapy (ATT) consisting of tablet isoniazid (INH) at 5 mg/kg, tablet rifampicin at 10 mg/kg, tablet pyrazinamide at 20 mg/kg, and tablet ethambutol at 15 mg/kg. Liver function tests (LFTs) were repeated after five days of therapy (Table 2).

**Table 2:** Laboratory Investigations done after five days of commencing anti-tubercular therapy INR: International normalized ratio; MELD-Na: model for end-stage liver disease-sodium

Parameter	Report	Normal limit
Hemoglobin	8.9	13.2-16.6gm/dL
Total leukocyte count	5300	4000-10,000/micro L
Platelet count	78000	1,50,000-4,10,000/micro L
Total bilirubin	2.1	0.22-1.2 mg/dL
Direct bilirubin	1.2	up to 0.5
Alanine transaminases	170	7-55IU/L
Aspartate transaminases	195	8-48 IU/L
Alkaline phosphatase	110	40-129 U/L
Serum Sodium	126	136-145 mmol/L
Serum Potassium	4.6	3.5-5.1 mmol/L
Serum Chloride	99	98-107 mmol/L
Serum protein	6.3	6.4-8.3 g/dL
Serum Albumin	2.4	3.5-5.2 g/dL
Prothrombin Time	16	10.09-13.79 seconds
INR	1.8	0.85-1.15
Serum Urea	36	17-49mg/dL
Serum Creatinine	1.2	0.6-1.35mg/dL
Child-Turcotte-Pugh score	Class C (10 points)	5-15 points
MELD-Na score	25 points	6-40 points

The patient was switched to a three-drug regimen comprising non-hepatotoxic drugs (injection streptomycin at 15 mg/kg, tablet levofloxacin at 15 mg/kg, tablet ethambutol at 15mg/kg) due to drug-induced liver injury, as the transaminases were elevated to more than three times the upper limit of normal. After seven days of the streptomycin, levofloxacin, and ethambutol (SLE) regimen, the patient’s LFTs were repeated, and they returned to the baseline. A trial with a low dose of tablet rifampicin at 150mg/day was initiated, and LFTs were monitored every seven days. The dose of tablet rifampicin was increased by 150 mg every seven days until a full dose of 450 mg was started for him after one month of beginning the SLE regimen. The patient’s CTP score remained constant at class B throughout anti-tubercular treatment with one hepatotoxic drug.

## Discussion

Individuals with liver cirrhosis who acquire TB are more often in a decompensated state, with CTP higher than grade A. While these patients have a higher chance of developing both pulmonary and extra-pulmonary tuberculosis, the incidence of extra-pulmonary manifestations, such as tuberculous peritonitis and disseminated tuberculosis, is higher compared to those without cirrhosis. The prevalence of multidrug-resistant tuberculosis is high in patients with chronic liver disease, and the *Mycobacterium* also demonstrates increased virulence in these patients [7].

Cirrhosis-associated immune dysfunction syndrome is a complex condition characterized by systemic immune disruption, the inability to remove bacteria, endotoxins, and cytokines from the bloodstream. Approximately 90% of the cells of the reticuloendothelial (RE) system, crucial for bacterial clearance, are housed in the liver, including sinusoidal endothelial cells and Kupffer cells. In patients with cirrhosis, factors such as portosystemic shunting and a reduction in the mass of RE cells result in bacteria and endotoxins bypassing the liver, allowing them to enter the systemic circulation more readily [7]. Neutrophil-mediated phagocytosis is also affected due to hyperammonemia and hyponatremia [9].

Managing TB in individuals with cirrhosis is a complex task that demands personalized approaches. The challenge arises from the hepatotoxic nature of the three primary anti-tubercular drugs (ATDs) and the vague diagnostic criteria for drug-induced hepatitis in cirrhotic patients. Rifampicin (RIF) is the least hepatotoxic, although it causes cholestatic jaundice. In contrast, pyrazinamide (PZA) is the most hepatotoxic anti-tubercular drug. In a meta-analysis, isoniazid (INH) demonstrated a higher likelihood of being linked to hepatotoxicity (odds ratio 1.6), even in the absence of RIF. Notably, when compared to each drug independently, the combination of INH and RIF was associated with a higher rate of hepatotoxicity (odds ratio 2.6) [8]. 

Despite the successful treatment of 85% of TB cases, significant morbidity arises from treatment-related adverse effects, including hepatotoxicity, hypersensitivity responses, and gastrointestinal and neurological diseases, thereby diminishing the efficacy of therapy. In 11% of patients receiving isoniazid, RIF, and pyrazinamide simultaneously, hepatotoxicity is the most common side effect that results in treatment cessation [8]. In our clinical practice, we discontinue hepatotoxic drugs if there is a relative increase in transaminases exceeding two times the baseline or if bilirubin rises above 2 mg/dl. Approximately 20% of patients taking isoniazid alone or in combination with other medications present with a transient, asymptomatic rise in liver enzyme levels that return to baseline with continued use [8]. When alanine transaminase (ALT) is at least three times the upper limit of normal (ULN) and there are reports of hepatitis symptoms and/or jaundice, or if ALT is at least five times the ULN without any symptoms, INH should be stopped. The majority of hepatitis develops four to eight weeks after medication begins. RIF can occasionally induce hepatocellular damage and increase the hepatotoxicity of other ATDs [7].

This emphasizes the importance of raising awareness among primary healthcare providers about the potential existence of undiagnosed chronic liver disease and the necessity for baseline liver function evaluation, especially in at-risk patients, before initiating first-line anti-tubercular treatment. The selection of regimens should be guided by the severity and instability of the liver condition, with an emphasis on reducing the number of hepatotoxic drugs as the liver disease progresses in severity or becomes more unstable. The CTP score serves as a crucial determinant in tailoring anti-tubercular treatment for individuals with varying degrees of liver disease. For patients with a CTP score of less than seven, indicating stable liver disease, the recommendation is to proceed with a regimen involving two potentially hepatotoxic drugs, with careful monitoring due to expected tolerance. However, for those in the advanced stage with a CTP score between eight and 10, the approach shifts to a regimen with only one hepatotoxic drug, favoring RIF over INH, while excluding PZA. In cases of very advanced liver disease with a CTP score of 11 or higher, the recommended treatment regimen excludes potentially hepatotoxic drugs. Instead, (kanamycin, ethambutol (EMB), streptomycin, amikacin, fluoroquinolones), and other second-line oral drugs are suggested for an extended period of 18-24 months. This stratified approach aligns treatment intensity with the severity of liver disease to optimize patient outcomes [7]. 

If the serum aspartate aminotransferase level exceeds three times the normal range before initiating treatment, and the abnormalities are not attributed to tuberculosis, various treatment options are available. One approach involves a six-month regimen with RIF, EMB, and PZA, excluding INH. Alternatively, a second option entails a nine-month treatment with INH, RIF, and EMB until the susceptibility to INH and RIF is confirmed, thereby excluding PZA. For patients with severe liver disease, a regimen comprising only one hepatotoxic agent, typically RIF plus EMB, may be administered for 12 months. Ideally, this should be complemented by another agent, such as a fluoroquinolone, during the initial two months of treatment [10]. 

In our patient, we observed a gradual improvement in the CTP scores. The periodic recalculation of CTP scores and the incorporation of the most effective anti-tubercular drugs, namely RIF and Isoniazid, hold significant importance. Fluoroquinolones, especially levofloxacin and ofloxacin, demonstrated both efficacy and safety when included in a hepatic-safe anti-tubercular treatment regimen for individuals with cirrhosis. However, the use of aminoglycosides such as streptomycin and amikacin is limited in cirrhosis due to the associated risk of acute kidney injury and coagulopathy.

## Conclusions

This case underscores the intricate challenges in managing TB in individuals with chronic liver disease with decompensation, emphasizing the need for tailored therapeutic strategies. The positive response to ATT, including the careful reintroduction of RIF, highlights the importance of personalized approaches and vigilant liver function monitoring. The case also emphasizes the imperative role of healthcare providers' awareness regarding the complex interplay between tuberculosis and liver cirrhosis, guiding optimal therapeutic decisions for improved patient outcomes. The broader perspective stresses the necessity of adopting a comprehensive and individualized approach, considering the specific condition of chronic liver disease, for the effective management of *Mycobacterium tuberculosis* infection.
